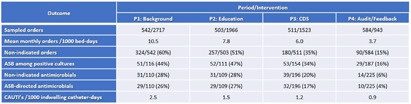# A Stepwise Diagnostic Stewardship Approach to Reduce Unnecessary Urine Cultures, Asymptomatic Bacteriuria, and CAUTI Rate

**DOI:** 10.1017/ash.2024.213

**Published:** 2024-09-16

**Authors:** Mina Said, Vanessa Kung, Abigail Beck, Deanna Buehrle, Kristin Nagaro, Graham Snyder, Elise Martin

**Affiliations:** Medicine / Infectious diseases UPMC; Pittsburgh VA Hospital; VA Pittsburgh; VA Pittsburgh Healthcare System; UPMC/University of Pittsburgh

## Abstract

**Background:** Clinically non-indicated asymptomatic bacteriuria (ASB) identification precipitates higher reported catheter-associated urinary tract infection (CAUTI) rates and urinary tract infection (UTI)-directed antimicrobial overuse. Published diagnostic stewardship interventions to reduce ASB were mostly tested individually and heterogeneously; hence the optimal bundle approach is yet to be defined. **Methods:** We performed a single-center sequential quasi-experimental study involving hospitalized, emergency, and long-term care patients at a VA healthcare facility, retrospectively comparing standard of care (period 1: 1/1/2022-6/30/2022) to adding dedicated provider education on facility-approved urine-culturing indications (period 2: 7/1/2022-1/19/2023), then adding an electronic clinical decision support (CDS) tool (Figure 1) mandating urine-culturing indications selections (period 3: 1/20/2023-6/30/2023), then prospectively adding real-time case-based physician-generated audit/feedback emails on ordering appropriateness (period 4: 7/1/2023-12/31/2023). We randomly sampled approximately 500 orders from each period and measured the impact on the rate of urine reflex/culture orders, the percentage of non-indicated orders and ASB, UTI-directed antimicrobial usage, and facility-wide CAUTI rates. **Results:** We analyzed 2140 urine reflex/culture orders (Table 1 and Figure 2). The mean monthly orders per 1000 bed-days and percentage of non-indicated orders decreased with each intervention to one-fourth of the initial values by period 4 (p=0.0002). The ASB rate among positive cultures was unchanged from periods 1 to 2 but started to decrease in period 3 with the biggest impact in period 4 (p=0.01). Non-indicated and ASB-directed antimicrobial courses both followed the previous pattern, dropping from 28% and 26% baseline to 6% and 4% by the study conclusion (p=0.015 and 0.008), respectively. Estimated UTI-directed antimicrobials decreased by 34% (363/551) with antimicrobial-days saved from 4093 to 2846 per 6-month period. CAUTI rate relatively declined with each intervention, along with a reduction in ASB-attributed CAUTI’s from 45% (5/11) initially to 20% (1/5) in period 4. **Conclusion:** A stepwise urine-culturing diagnostic stewardship approach of clinical education, electronic CDS tool, plus real-time audit/feedback decreased overall urine reflex/cultures, non-indicated ordering, ASB identification, unnecessary antimicrobials, and CAUTI rates, with the greatest impact after bundling all interventions including order appropriateness audit/feedback.

**Figure:**
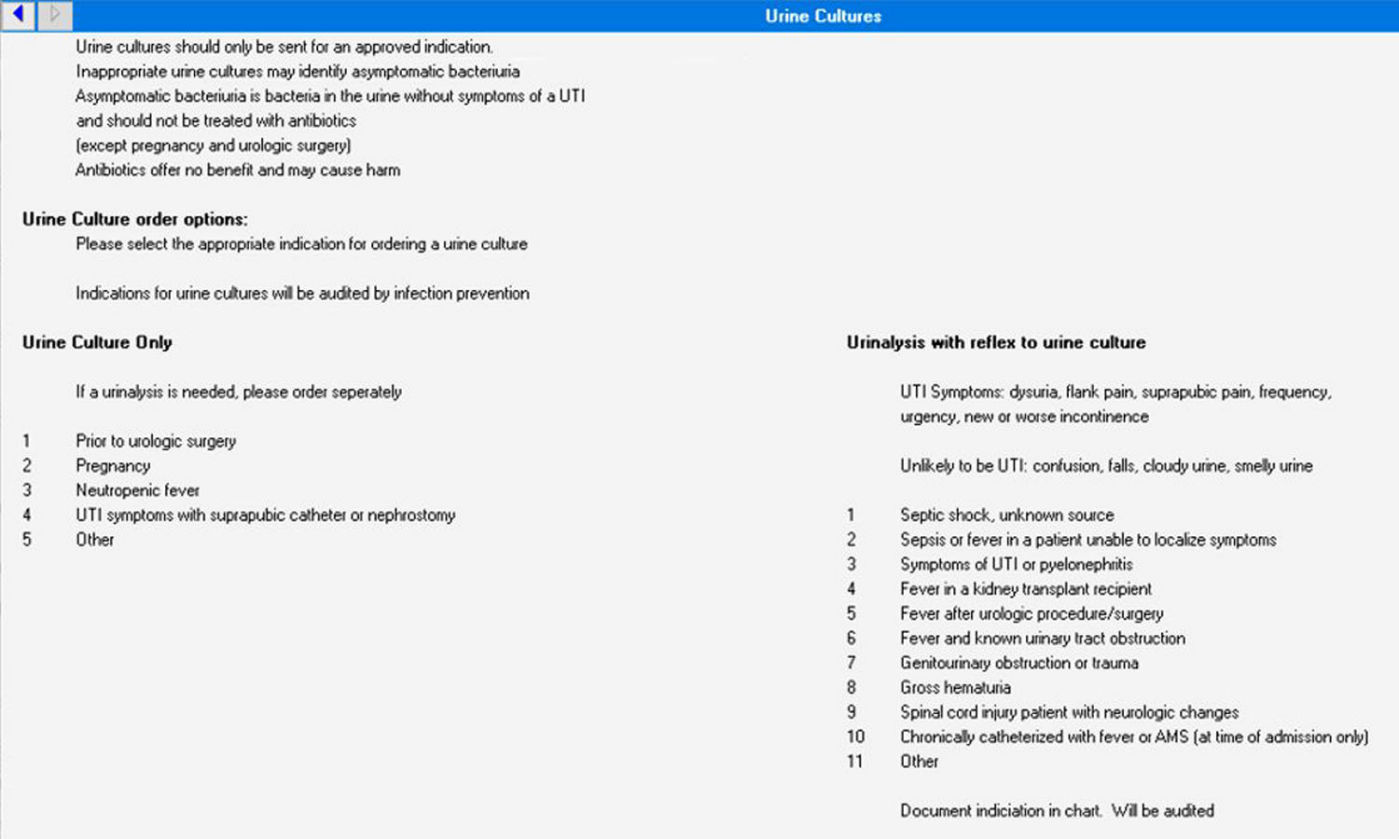

**Figure:**
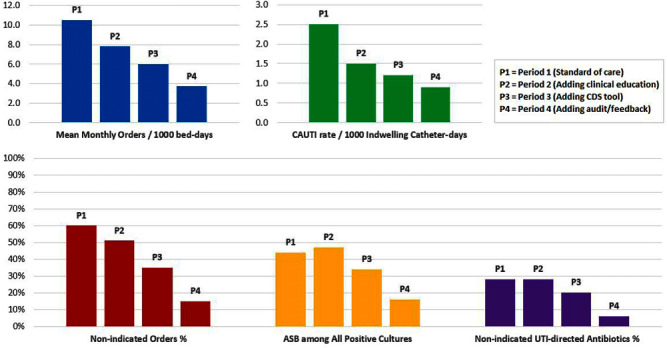

**Figure:**